# Study on Intrinsic Influence Law of Specimen Size and Loading Speed on Charpy Impact Test

**DOI:** 10.3390/ma15113855

**Published:** 2022-05-28

**Authors:** Wang Jia, Aiguo Pi, Zhang Zhao, Shaohong Wang, Chen Wei, Zhou Jie, Fenglei Huang

**Affiliations:** State Key Laboratory of Explosion Science and Technology, Beijing Institute of Technology, Beijing 100081, China; wangjiafy@foxmail.com (W.J.); nash13zhao@gmail.com (Z.Z.); aimee_wsh@163.com (S.W.); wi_chan@163.com (C.W.); zhoujiepla@foxmail.com (Z.J.); huangfl@bit.edu.cn (F.H.)

**Keywords:** 30CrMnSiNi2A, Johnson-Cook (J-C) model, Charpy impact test, impact toughness, sub-size specimens, constitutive model

## Abstract

Charpy impact energy/impact toughness is closely related to external factors such as specimen size. However, when the sample size is small, the linear conversion relationship between the Charpy impact energy of the sub-size and full-size Charpy specimens does not hold; the Charpy impact toughness varies with the size of the specimen and other factors. This indicates that studying the internal influence of external factors on impact energy or impact toughness is the key to accurately understanding and evaluating the toughness and brittleness of materials. In this paper, the effects of strain rate on the flow behavior and the effects of stress triaxiality on the fracture behavior of 30CrMnSiNi2A high-strength steel were investigated using quasi-static smooth bar and notched bar uniaxial tensile tests and Split Hopkinson Tensile Bar (SHTP). Based on the flow behavior and strain rate dependences of the yield behavior, a modified JC model was established to describe the flow behavior and strain rate behavior. Charpy impact tests were simulated using the modified JC model and JC failure model with the determined parameters. Reasonable agreements between the simulation and experimental results have been achieved, and the validity of the model was proved. According to the simulation results, the impact energy was divided into crack initiation energy, crack stability propagation energy and crack instability propagation energy. On this basis, the effects of striker velocity and specimen width on the energy and characteristic load of each part were studied. The results show that each part of the impact energy has a negligible dependence on the hammer velocity, but there is a significantly different positive linear relationship with the width of the sample. The energy increment of each part also showed an inverse correlation with the increase in the sample width. The findings reveal that the internal mechanism of Charpy impact toughness decreases with the increase in sample width; to a certain extent, it also reveals the internal reason why the linear transformation relationship of Charpy impact energy between sub-size specimens and standard specimens is not established when the specimens are small. The analytical method and results presented in this paper can provide a reference for the study of the dynamic behavior of high-strength steel, the relationship between material properties and sample size, and the elastic–plastic impact dynamic design.

## 1. Introduction

30CrMnSiNi2A alloy steel is a high-strength low-alloy steel that is widely used in the manufacture of aircraft landing gear, wings, engines, shells, and other aviation structural parts. Owing to the complexity of the operating environment, steel members often experience one or more large energy shocks [1,2]. The impact resistance of materials is usually evaluated by Charpy impact energy. Therefore, when selecting materials for structural design, the impact resistance of materials is as important a performance index as the strength of materials [3,4,5]. However, a large number of studies have shown that the variation law of impact energy with specimen size is not linear, and anomalies occur at small sizes [6,7,8,9,10,11,12]. Meanwhile, impact toughness decreases with the increase in specimen size [6,10,13]. Furthermore, the impact energy includes crack initiation energy, crack stability propagation energy and crack instability propagation energy [14]. Most energy is consumed in the crack initial stage [5,11,15]. The crack initiation energy and crack stability energy are the key part to evaluating the impact resistance of materials [11]. The proportion of each part of different materials in the impact energy is quite different [11,14]. All these indicate that the overall impact energy cannot accurately understand and evaluate the ductility and brittleness of the material, and it cannot reveal the variation law of the ductility and brittleness of the material with the width of the sample. Therefore, it is very meaningful and necessary to divide the impact energy and study the influence of various factors on the energy of each part.

Through the instrumented impact testing machine, information such as the load–displacement curve and energy–displacement curve during the test can be obtained [6,16]. Based on those information, we can obtain a preliminary understanding of the energy distribution of each part in the impact energy and divide the impact energy according to the load–displacement curve, allowing us to deeply study the impact resistance of the material. Many scholars through experiments and theoretical analysis have proposed different views about the crack initiation position and divided the impact energy into different parts, such as: the load plummeting [5], max load point [17,18,19], the (P_max_ + P_GY_)/2 (P_max_ is the maximum load and P_GY_ is the yield load) [20], or at about 80% of the maximum load energy [11,15,21,22,23], etc. Although many scholars have proposed different views on the divide of impact energy, no unified conclusion has been reached, and it is still impossible to study the influence of different factors on the energy of different parts of the impact energy. With the development of finite element software, a material constitutive model and fracture model research, more and more researchers are paying more and more attention to the simulation research on the Charpy impact test and have achieved good results [12,24,25,26,27]. However, the researchers did not conduct in-depth research on the distribution of the energy of each part in the impact energy according to the simulation results, which is insufficient.

The constitutive model and fracture model of materials are the keys to accurately simulating the Charpy impact test. The Johnson–Cook (JC) model is simple in form and allows an easy determination of parameters; it can accurately describe the deformation behavior of materials at high temperature, high strain, and high strain rate [28], and it is widely used to study metal impact or similar problems. Numerous studies have shown that the JC model and JC fracture model can accurately predict the flow behavior of metals under dynamic conditions. Banerjee [29] et al. determined the model parameters of the JC model of typical armor steel materials through experiments, used the determined parameters to simulate the Charpy impact test of armor steel, and obtained reliable prediction results. Cao [12] and Chen [24] determined the parameters of the JC constitutive model and JC fracture model through experiments, simulated the Charpy impact test of X80 pipeline steel and 6082-T6 alloy aluminum, respectively, and ascertained that the fracture morphology, impact energy and the load–displacement curve are in good agreement with the experiment results, which proves the validity of using the JC constitutive model and JC fracture model to study the Charpy impact problem. Although many simulation studies have been carried out on the Charpy impact test, they have not investigated the proportion of each part of the energy in the impact energy based on the comparative analysis of the simulation and test results, nor have they studied the change law of the relationship between the energy of each part and external factors, which is insufficient obviously.

Furthermore, the Charpy impact test results are affected by many factors, and scholars have conducted many studies in this area. Madhusudhan [30] and Cao [12] studied the effect of several different striker velocities on the Charpy impact test of 300 maraging steel and X80 pipeline steel using ABAQUS software. In addition, it has important research values to transform non-standard test results into standard test results. To establish the relationship between the Charpy impact energy of the standard full-size Charpy specimen (CVN) and the sub-size Charpy specimen (SCVN), many scholars [6,7,8,9,10,11,12] have conducted numerous experiments and simulation studies. The ASTM A370-2019 [31] standard stipulates that the conversion relationship between them is proportional to width. However, some studies found that this rule is invalid when the sample size is small, and it is insufficient to reveal the internal reason why the linear relationship is invalid.

In conclusion, the comprehensive and systematic study of the Charpy impact test by combining experiments and simulations has become an effective method to study the Charpy impact test of auxiliary materials. The impact energy can be divided according to the simulation results. The proportion of each part of the impact energy and its variation with external factors can be studied in detail using this approach. Some important problems, such as the conversion relationship of impact energy with different widths and the impact toughness decreasing with the increase in sample size, can be analyzed and discussed to reveal the internal mechanism. It has higher research significance and value.

In this paper, the effects of strain rate and stress state on the mechanical behavior and fracture behavior of 30CrMnSiNi2A high-strength steel were investigated through a large number of experiments. First, the effect of strain rate on material flow behavior was studied using smooth bar specimens under quasi-static uniaxial tensile tests and SHTP tests, A modified Johnson–Cook (MJC) model was established, the parameters of the MJC model and JC failure model were determined, and the VUMAT subroutine was developed based on the ABAQUS software. The Charpy impact test was simulated based on the determined parameters. According to the simulation and experiment results, the accuracy of the finite element model and the MJC model was verified. Furthermore, the impact energy was divided into the crack initiation energy, crack stability energy and crack instability energy. Finally, based on the MJC model parameters and JC damage model parameters combined with the finite element method, the influence of the striker velocity and sample width on the energy and characteristic load of each part of the Charpy impact test of 30CrMnSiNi2A high-strength steel was comprehensively analyzed. The internal reasons why the linear conversion relationship of impact energy of different sizes does not hold for small-size specimens are discussed, and the mechanism that the impact toughness decreases with the increase in specimen size is also analyzed. Finally, a correlation formula of the energy and the characteristic load of each part of the Charpy impact test between CVN and SCVN is established.

## 2. Experimental and Methods

### 2.1. Experimental Design

**Figure 1:**
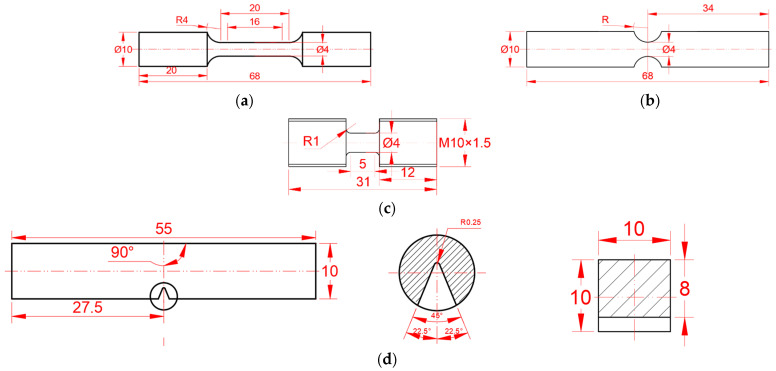
Geometry and dimensions (in mm) of each type of specimen. (**a**) Static smooth round tensile bar [32]; (**b**) Notched round tensile bar (R = 3, 4, and 9 mm) [12]; (**c**) Dynamic round tensile bar [33]; (**d**) Standard Charpy sample [12].

The chemical composition of the 30CrMnSiNi2A high-strength steel used in this study is listed in Table 1. To study the influence of different strain rates and stress states on mechanical behavior and fracture behaviors, the quasi-static smooth and notched bar and dynamic tensile bar specimens were designed according to [32], as shown in Figure 1a–c. Tensile tests of quasi-static smooth and notched bars were carried out by the MTS machine. A dynamic tensile test was carried out by the Split Hopkinson Tensile Bar (SHTB). To study the impact properties, the standard Charpy V-notch samples were designed according to [12], as shown in Figure 1d. The impact test was carried out by an instrumented impact testing machine. To ensure repeatability and consistency, three samples were tested for each case.

The section diameter of the smooth bar specimens was 4 mm, and the lengths of the quasi-static and dynamic test sections were 16 and 5 mm, respectively. The section diameters of the notched bar samples are 4 mm, and the notched radii were 3, 4, and 9 mm, respectively. The Charpy V-type sample length was 55 mm, the width and height were 10 mm, and the notch depth was 2 mm. 

### 2.2. Experimental Methods and Results

#### 2.2.1. Static Smooth and Notched Test

**Figure 2:**
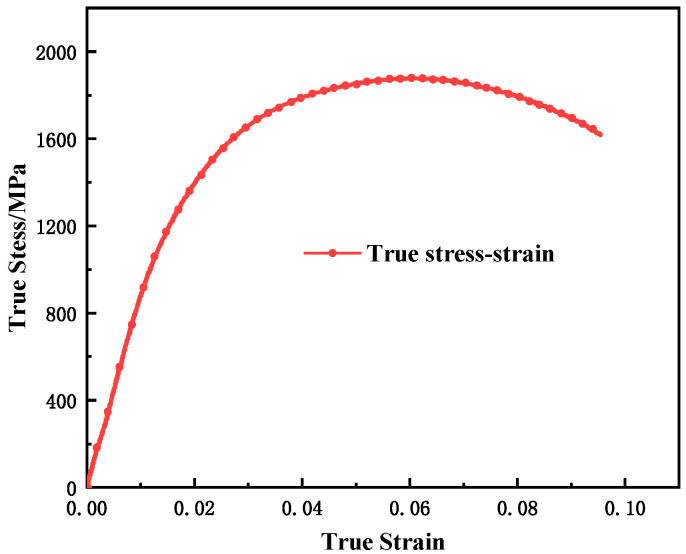
Static smooth tensile test results (0.001 s^−1^).

The smooth round bar quasi-static uniaxial tensile tests were carried out at room temperature using MTS machine, and the strain rates were 0.001 s^−1^ and 0.1 s^−1^, respectively. The strain rate and engineering strain were calculated using Formulas (1)–(3), respectively, where $l_{0}$ denotes the effective length of the specimen, $A_{0}$ denotes the cross-sectional area of the test section, $\dot{\varepsilon }\left(t\right)$ denotes the strain rate, V(t) denotes the loading speed, $\;F_{a}\left(t\right)$ denotes the force, and $U_{relative}\left(t\right)$ denotes the displacement. The notched tensile test was also carried out with MTS under the loading conditions of the smooth bar quasi-static tensile test, with notch radii of 3, 4, and 9 mm. The true stress–strain curves are shown in Figure 2. The material has no strict linear elastic segment, and $\sigma_{0.2}$ is used as the yield strength of the material. The mechanical properties of the static smooth bars under tensile conditions are shown in Table 2.

(1) $$\dot{\varepsilon }\left(t\right)=V\left(t\right)/l_{0}$$

(2) $$\sigma \left(t\right)=F_{a}\left(t\right)/A_{0}$$

(3) $$\varepsilon \left(t\right)=U_{relative}\left(t\right)/l_{0}$$

#### 2.2.2. Dynamic Tensile Test

**Figure 3:**
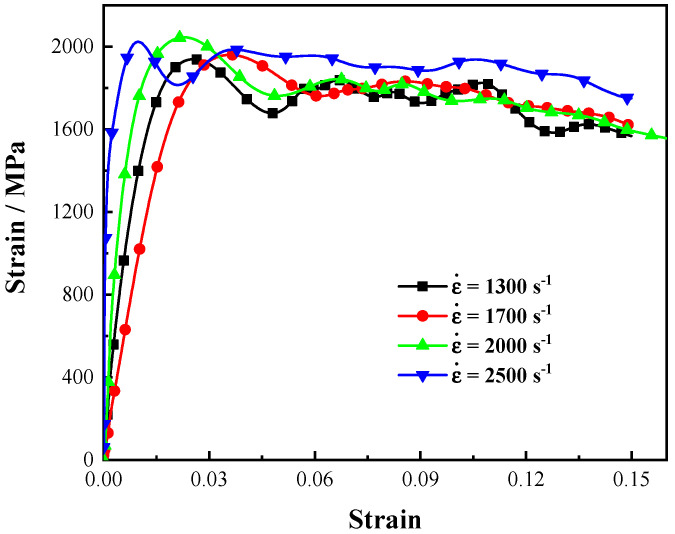
True stress–strain curves of SHTB tensile test at different strain rates.

Dynamic tensile tests were carried out by SHTP at room temperature. The SHTP device consists of a striker bar, an incident bar, and a transmitter bar, all of which have a diameter of 19 mm. In the tests, based on one-dimensional elastic stress wave theory, the force and displacement were determined according to the strain gauge signals collected on the incident and transmitter bar, and then, the stress and strain information of the tested sample was obtained according to the geometric size of the sample. For example, for compression tests,$\;\varepsilon_{i}\left(t\right)$, $\varepsilon_{r}\left(t\right)$, and $\varepsilon_{t}\left(t\right)$ represent the incident, reflected, and transmitted waves, respectively. The stress, strain, and strain rate of the compression sample can be calculated by Equation (4), where E and C, respectively, represent the Young’s modulus and longitudinal wave velocity of the incident and transmitter bars, respectively, $L_{0}$ represents the initial length of the test part of the sample; and $A/A_{s}$ represents the ratio of the cross-sectional area of the bar to the sample. Loading under different strain rates can be performed by controlling the striker velocity using air pressure. The dynamic tensile mechanical properties under different strain rates were obtained, as shown in Figure 3.

(4) $$\{\begin{array}{c} \sigma \left(t\right)=E\left(A/A_{s}\right)\varepsilon_{t}\left(t\right)\\ \varepsilon \left(t\right)=-\left(2C/L_{0}\right)\int_{0}^{t}\varepsilon_{r}\left(t\right)dt\\ \dot{\varepsilon }\left(t\right)=-\left(2C/L_{0}\right)\varepsilon_{r}\left(t\right) \end{array}$$

#### 2.2.3. Charpy Impact Test

The Charpy impact tests were performed on the instrumented impact testing machine according to the ASTM E23 standard. The nominal energy of the striker impact testing machine was 300 J. The test device consisted of a hammer with a mass of 21.9 kg, a data acquisition instrument, and a computer; the span length between the anvils was 40 mm.

The experiment was carried out at room temperature without considering the effect of temperature. The striker with a radius of 2 mm was selected. The sample was placed on the anvil with centering pliers to ensure that the center between the anvil and the centerline of the sample gap coincided. The strike speed was 5.24 m/s. Bearing friction and air resistance were ignored when calculating the energy absorbed by the sample. The data acquisition instrument synchronously collected data during the test.

## 3. Material Model and Parameter Determination

### 3.1. JC Model

Based on continuous damage mechanics and viscoplastic mechanics, Johnson and Cook comprehensively analyzed the effects of high strain rate, temperature, and large deformation. Based on von Mises yield criterion, they postulated the multiplicative decomposition of flow stress into three functions that solely depend on the strain, strain rate and temperature; and they proposed the JC model (Equation (5)), which includes linear elastic yield, plastic flow, isotropic strain hardening, strain rate hardening, thermal softening and other factors [12,24,29]. In addition, this model can predict the flow behavior of materials [28].

(5) $$\sigma_{eq}=\left(A+B\varepsilon_{p}^{n}\right)\left(1+\mathrm{Cln}{\dot{\varepsilon }}^{*}\right)\left(1-T^{*}{}^{m}\right)$$

where *A*, *B*, *n*, *C,* and *m* are material parameters, *A* is the yield stress of the material at the reference strain rate and temperature, and *B* and *n* are the strain-hardening modulus and strain-hardening index of the material, respectively. *A*, *B*, and *n* can be obtained by a quasi-static test at the reference temperature. *C* is the strain rate-hardening coefficient, which can be obtained by dynamic tests under different strain rates. *m* is the thermal softening coefficient, which can be obtained by calibrating the unidirectional tensile test data at different strain rates and temperatures. $\sigma_{eq}$ is the equivalent stress, $\varepsilon_{p}$ is the plastic strain, ${\dot{\varepsilon }}^{*}=\dot{\varepsilon \;}/{\dot{\varepsilon }}_{0}$ is the dimensionless strain rate, ${\dot{\varepsilon }}_{0}$ is the reference strain rate, $\dot{\varepsilon \;}$ is the strain rate, $T^{*}=\left(T-T_{0}\right)/\left(T_{m}-T_{0}\right)\;$is the dimensionless temperature; T, $T_{0}$, and $T_{m}$ are the operating temperature, reference temperature, and metal melting point, respectively. Because the effect of temperature was not considered in this study, this item was ignored. Therefore, Equation (4) can be simplified to Equation (6) as follows:

(6) $$\sigma_{eq}=\left(A+B\varepsilon_{p}^{n}\right)\left(1+\mathrm{Cln}{\dot{\varepsilon }}^{*}\right)$$

According to the preliminary analysis of the experimental results, the original JC model could not accurately describe the flow behavior and stress rate behavior of 30CrMnSiNi2A high-strength steel, and a modified JC model (MJC model) was established.

### 3.2. MJC Model

#### 3.2.1. Modification of Strength Part

**Figure 4:**
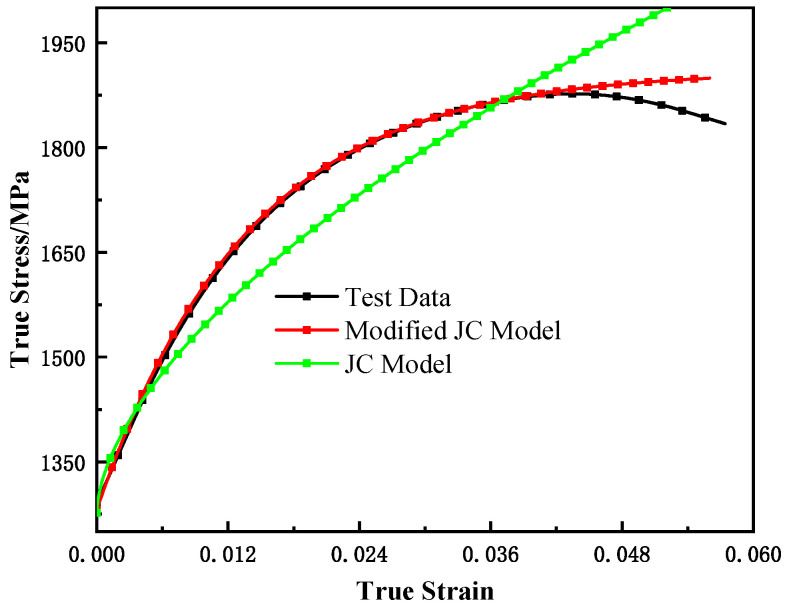
The comparison of model predictions with experimental results at strain rate 0.001 s^−1^.

Based on the quasi-static smooth round bar uniaxial tensile test results, the original JC model (Equation (7)) cannot accurately predict the flow behavior of the material. In our modified model, the Ludwik hardening criterion [33] in the original JC model is replaced by the Voce hardening criterion [34]. This model modification was inspired by the works of Sung et al. [35] and Mohe et al. [36,37,38,39], too. The corrected flow stress expression is given by Equation (8). According to the test results, the parameters of the strength term are determined as E = 70.75 GPa, A = 1290 MPa, B = 595 MPa, w = 1.084, and *n* = 0.01435. As shown in Figure 4, the revised model can predict the flow behavior of the material more accurately.

(7) $$\sigma_{eq}=\left(A+B\varepsilon_{p}^{n}\right)$$

(8) $$\sigma_{eq}=A+B\left(1-w*\exp\left(-\varepsilon_{p}/n\right)\right)$$

#### 3.2.2. Modification of Strain Rate

**Figure 5:**
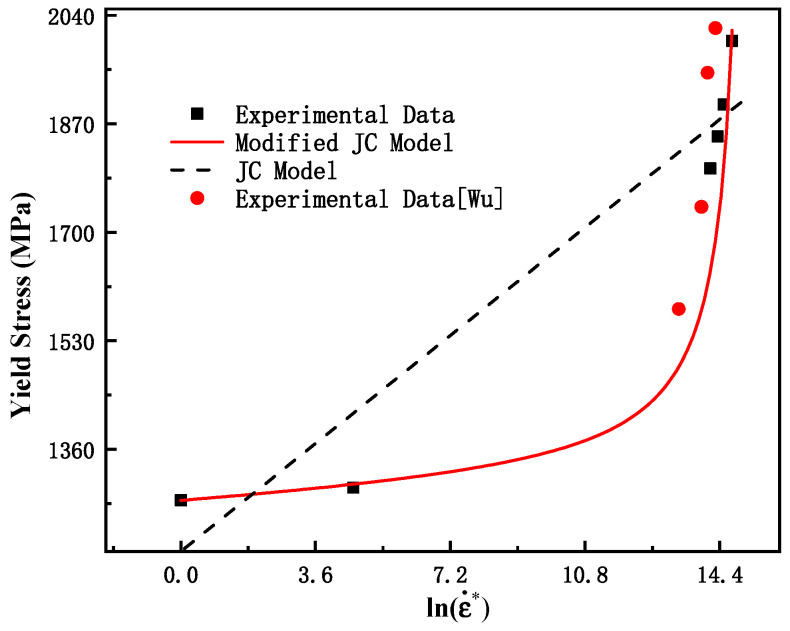
Variation of yield stresses with logarithmic strain rates.

To further understand the effect of the strain rate on the yield stress, Figure 5 plots the yield stress vs. strain rate at room temperature. As seen in this figure, the strain rate increases from 0.001 s^−1^ to 0.1 s^−1^, the yield stress shows a slow increase with the increase in $ln{\dot{\varepsilon }}^{*}$, and when the strain rate increases further, the yield stress increases rapidly. According to the JC model, the yield stress increases linearly with the increase in $ln{\dot{\varepsilon }}^{*}$. The yield stress of many other ductile metals also shows a huge increase when the strain rate exceeds 10^2^ s^−1^ or 10^3^ s^−1^ [40,41,42]. Therefore, the original JC model (Equation (9)) cannot describe the dependence of yield stress on the strain rate, as shown in Figure 5. In order to improve the accuracy of the model prediction in the high strain rate range, the strain rate term of the original JC model was corrected, and the modified JC strain rate term can be expressed as shown in Equation (10). To improve the accuracy of the model fitting, the dynamic tensile test data of Wu [43] were also used simultaneously. The fitting results were: *C*_1_ = 0.0025, *C*_2_ = 0.029, and *C*_3_ = 15.25. Figure 5 shows the prediction results of the test. As shown, that the modified model can better describe the dependence of the yield stress on the strain rate of the material studied in this paper.

(9) $$\sigma_{eq}=A\left(1+\mathrm{Cln}{\dot{\varepsilon }}^{*}\right)$$

(10) $$\sigma_{eq}=A\left(1+C_{1}ln{\dot{\varepsilon }}^{*}+C_{2}\left(\frac{1}{C_{3}-ln{\dot{\varepsilon }}^{*}}-\frac{1}{C_{3}}\right)\right)$$

where *C*_3_ is the natural logarithm of the critical strain rate level and *C*_1_ and *C*_2_ are the material parameters. For the convenience of parameter expression, the reference strain rate was 0.001 s^−1^, and the reference temperature was room temperature.

#### 3.2.3. Model Validation

**Figure 6:**
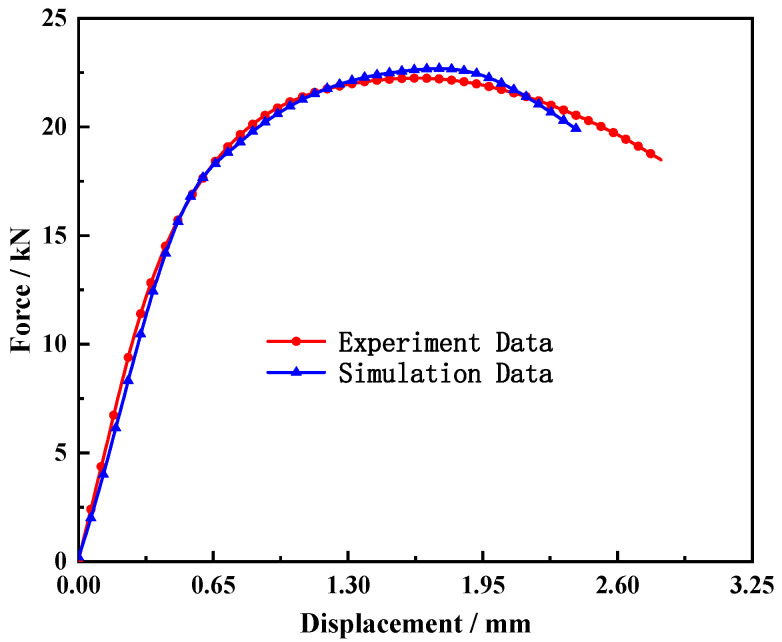
Comparison of load–displacement between simulation and smooth bar tensile test results (0.001 s^−1^).

**Figure 7:**
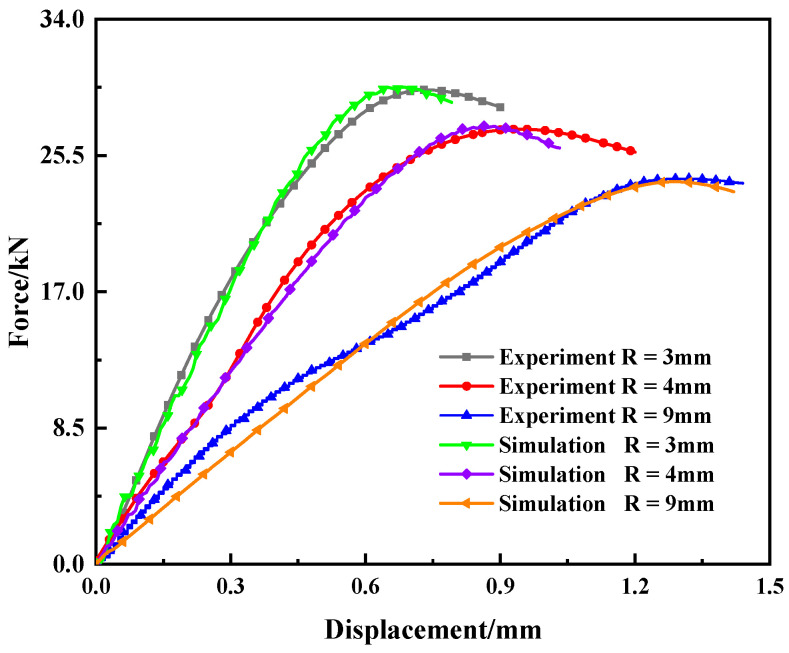
Comparison of load–displacement between simulation and notched bar tensile test results (R = 3, 4, 9 mm).

A VUMAT subroutine was developed based on the ABAQUS simulation software. To verify the validity of the MJC model and the determined parameters, the quasi-static tensile tests of the smooth and notched bars were simulated, and the results are shown in Figure 6 and Figure 7. The validity and accuracy of the MJC model, determination of the parameters, and subroutines were proved.

### 3.3. Johnson–Cook Failure Model

The failure model proposed by Johnson–Cook considers the dependence of the plastic fracture strain $\varepsilon_{f}$ on the stress triaxiality, strain rate, and temperature [28], as shown in Equation (11). It is assumed that damage accumulates in the material element during plastic straining, which accelerates immediately when the damage reaches a critical value.

(11) $$\varepsilon_{f}=\left(D_{1}+D_{2}\exp\left(D_{3}\sigma^{*}\right)\right)\left(1+D_{4}\ln{\dot{\varepsilon }}^{*}\right)\left(1+D_{5}T^{*}\right)\;$$

*D*_1_–*D*_3_ are material damage parameters dependent on stress triaxiality. *D*_4_ is a material damage parameter that is dependent on the strain rate. *D*_5_ is a material damage parameter that is dependent on temperature. $\sigma^{*}$ is the triaxiality of the stress. The temperature term is not considered in this study; therefore, only *D*_1_–*D*_4_ need to be determined.

#### 3.3.1. Determination of Damage Parameters *D*_1_, *D*_2_, and *D*_3_

Under the conditions of reference strain rate and temperature, the JC failure model can be simplified as Equation (12).

(12) $$\varepsilon_{f}=\left(D_{1}+D_{2}\exp\left(D_{3}\sigma^{*}\right)\right)$$

*D*_1_–*D*_3_ can be determined by quasi-static smooth and notched round bar tensile tests at reference strain rates and temperature. The first method involves substituting the geometric dimensions of the specimen at fracture into Equation (13), as proposed by Bridgman [44].

(13) $$\sigma^{*}=1/3+\ln\left[1+r/\left(2R\right)\right]$$

**Figure 8:**
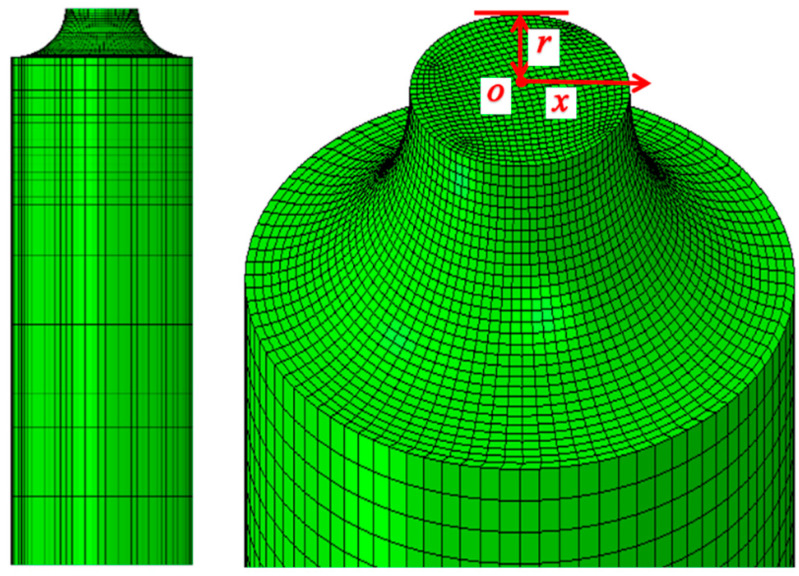
Schematic diagram of the minimum cross-section of the notched round bar specimen.

*R* and *r* are the radii of the curvature of the gap and the minimum cross-section of the specimen, respectively. Due to the deformation of the sample during the test, the radius of the sample changed, and the Bridgman’s formula was no longer applicable. A more accurate method for determining the stress triaxiality is to calculate it according to Equation (14) based on numerical simulation results [45].

(14) $$\sigma^{*}=\sigma_{H}/\sigma_{eq}$$

where $\sigma_{m}$ and $\sigma_{eq}$ are the hydrostatic pressure and von Mises equivalent stress, respectively. The distribution of stress triaxiality on the minimum cross-section of the notched round specimen was extracted based on the simulation results. *x* is defined as the distance from a point to the center of the section, as shown in Figure 8.

**Figure 9:**
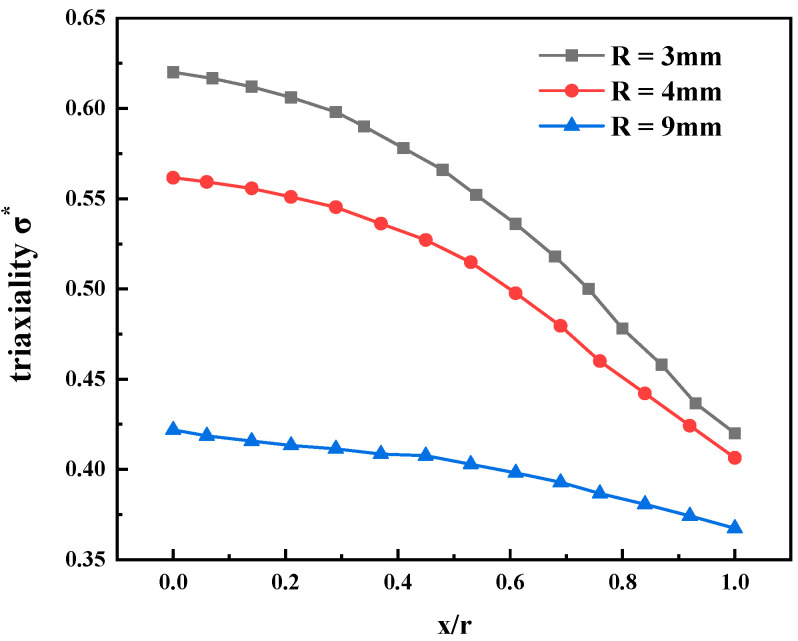
Distributions of stress triaxiality at the minimum section of notched round bar specimens with different notch radii.

The distribution of stress triaxiality was not uniform throughout the section, as shown in Figure 9. The stress triaxiality of the samples with a different notch radii was close to that of the free surface of the minimum section. The smaller the notch radius is, the closer it is to the sample center, and the greater the stress triaxiality. For specimens with different notch radii, the maximum stress triaxiality is located at the center of the minimum section, that is, *x/r* = 0, the stress triaxiality then decreases with the increase in the *x/r* value. The center position of the minimum section of the notched sample was selected to determine the stress triaxiality, as shown in Table 3.

After the stress triaxiality is determined, the fracture strain of the notched bar specimen needs to be further determined. The fracture strain value, $\varepsilon_{f}$, can be measured by the change of the minimum cross-section diameter of the smooth and notched tensile bar before and after the test. It can be calculated using the method of Hancock [46], which is given by Equation (15).

(15) $$\varepsilon_{f}=2\ln\left(r/r_{f}\right)$$

where *r* is the initial radius of the minimum cross-section, and$\;r_{f}$ is the fractured radius of the minimum cross-section. Owing to the simplicity of the formula, the ease of calibration, and wide applicability to many metal materials [12,24], then this method is widely used.

**Figure 10:**
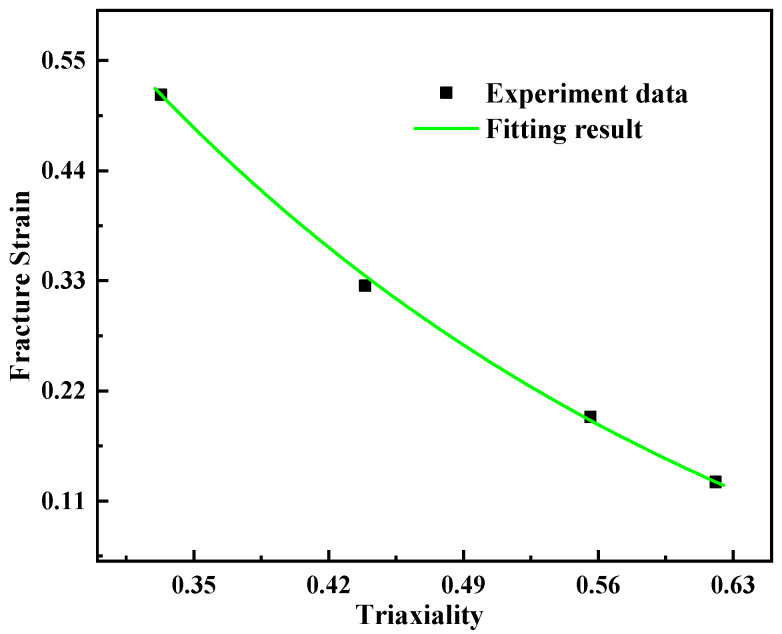
Relationship between stress triaxiality and fracture strain.

The fitting curve is presented in Figure 10. Correspondingly, the failure material parameters can be determined as $D_{1}=-0.1663$, $D_{2}=1.7969$, and $D_{3}=-2.9078$.

#### 3.3.2. Determination of Damage Parameter *D*_4_

Next, the parameter *D*_4_, which depends on the strain rate, needs to be determined. This parameter can be obtained by performing dynamic tensile tests at different strain rates. In this study, to better simulate the Charpy impact test, Cao’s [12] method is adopted; that is, *D*_4_ is determined by comparing the results between the Charpy impact test and those of the numerical simulation.

**Figure 11:**
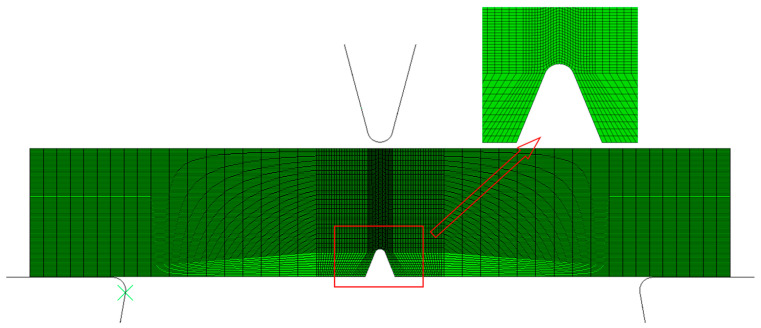
FE model of Charpy impact test.

The finite element model of the Charpy impact test is established, as shown in Figure 11. The model consists of three parts: the striker, the anvils, and the specimen. The simulation design is consistent with the test. Striker and anvils are set as rigid bodies, and the sample is variable. The mesh at the notch region is refined, and the remaining part of the specimen is coarsened. The minimum mesh size is 0.02 mm. The element type is the three-dimensional hexahedral element with reduced integration (C3D8R). According to the test condition, the boundary of the sample is constrained by arresting movement along the x-and y-direction, and the striker falls only in the y-direction at the velocity of 5.24 m/s. The kinematic contact between specimen and striker was assigned with surface to surface control interface with frictional interaction.

**Figure 12:**
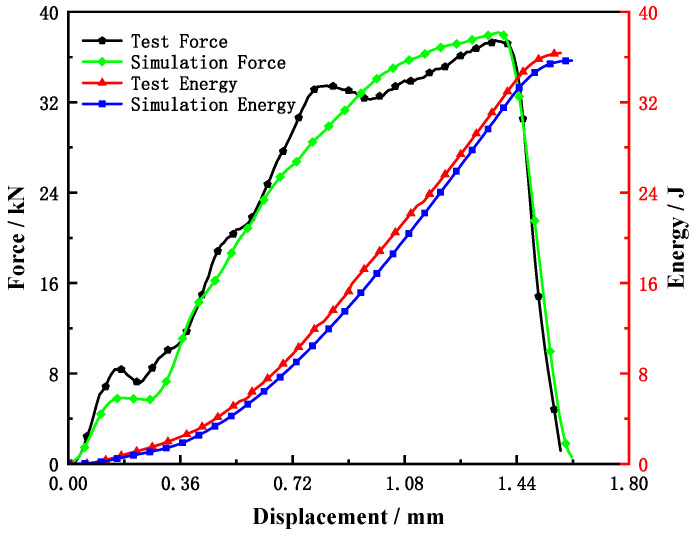
Comparison of force vs. displacement and energy vs. displacement between simulated and experimental Charpy impact tests.

**Figure 13:**
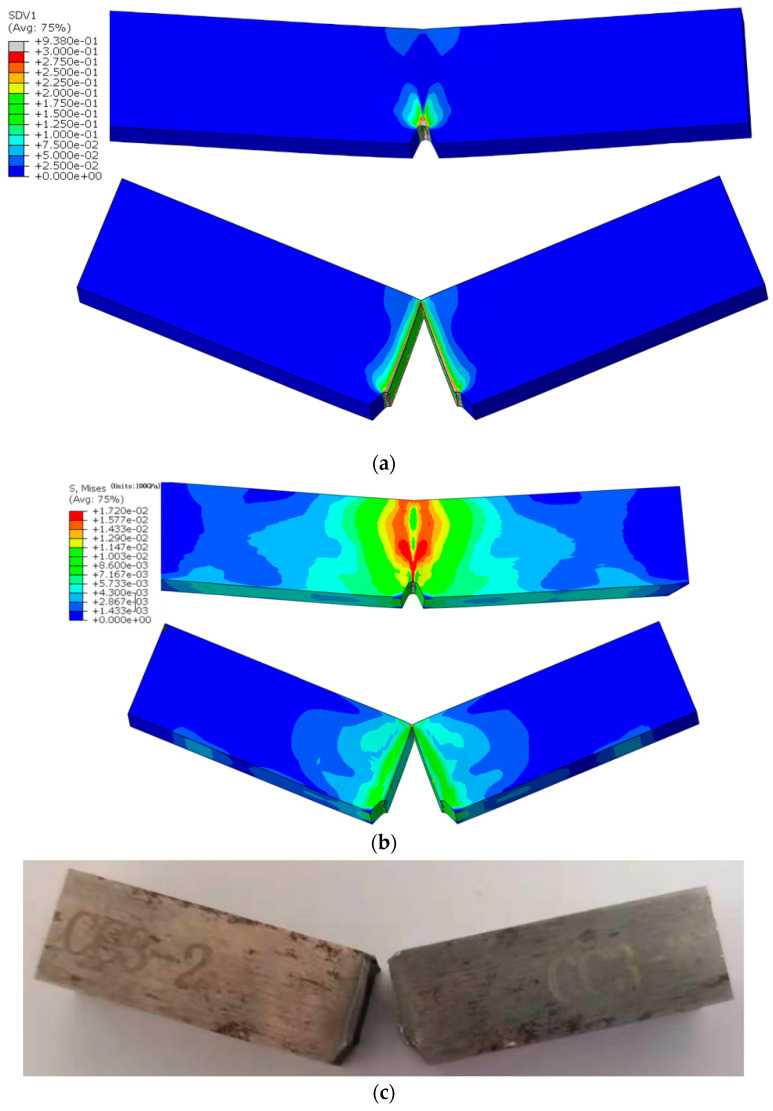
Comparison of deformation morphology between simulation and experimental samples; (**a**) Distribution of equivalent plastic strain (SDV1); (**b**) Distribution of Mises stress; (**c**) Experiment fracture.

According to the determined MJC model parameters (*E* = 70.75 GPa, *A* = 1315 MPa, *B* = 595 MPa, *w* = 1.084, *n* = 0.01435) and some damage parameters of the JC fracture model (*D*_1_ = −0.1663, *D*_2_ = 1.7969, *D*_3_ = −2.9078), parameter *D*_4_ is adjusted until the load–displacement curves of the simulation and experiment are consistent, and then, the parameter *D*_4_ is determined. Through calculation, it is found that when *D*_4_ = 0.07, the differences between the maximum load and impact energy obtained by the test and simulation are 1.98% and −1.79%, respectively, as shown in Table 4; and as well as Figure 12 and Figure 13, which indicates that the simulation results are in good agreement with the experiment results. The results shows that the MJC model, determined model parameters and damage parameters are effective and feasible to simulate the Charpy impact test. It can be seen from Figure 13a,b that the plastic strain and Mises stress of the sample is mainly distributed near the root of the notch, and the crack is generated from the center of the notch and extends through the sample.

## 4. Results and Discussion

The Charpy test results are affected by many factors, such as the state of the specimen (size, notch type, notch depth, etc.), the state of the impact testing machine (stiffness, hammer radius, etc.), test conditions (impact velocity, temperature, etc.), sample material inhomogeneity, operator differences, etc. Studying the effects of striker velocity and specimen width on Charpy impact results has always been an important focus of research [6,7,8,9,10,11,12,30]. In this paper, the striker velocity and the width of the sample are selected to study the influence of two factors.

**Figure 14:**
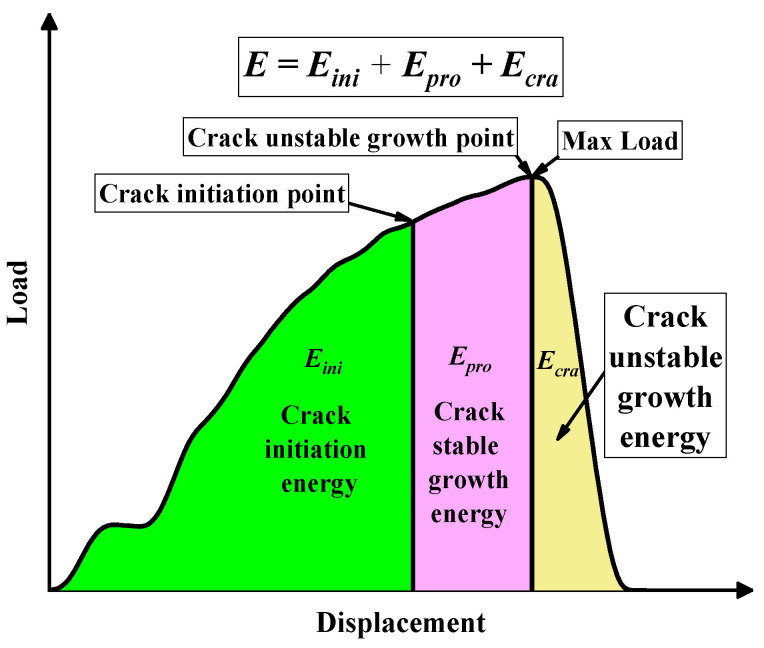
Schematic diagram of the impact energy division.

To study the effect of the striker velocity and sample size on the Charpy test results in detail, the accuracy of the simulation model was first verified according to the load–displacement curve and energy–displacement curve of the test, as shown in Figure 12. Then, the Charpy impact tests with different striker speeds and sample widths were simulated and studied, and then, the crack initiation point and crack instability propagation were determined according to the simulation results point to divide the impact energy. The energy before the crack initiation point is the crack initiation energy, the energy between the crack initiation point and the crack instability propagation point is the crack stable propagation energy, and the remaining energy is the crack instability propagation energy. The division of the energy absorbed by the sample is shown in Figure 14.

### 4.1. Effect of Striker Velocity

**Figure 15:**
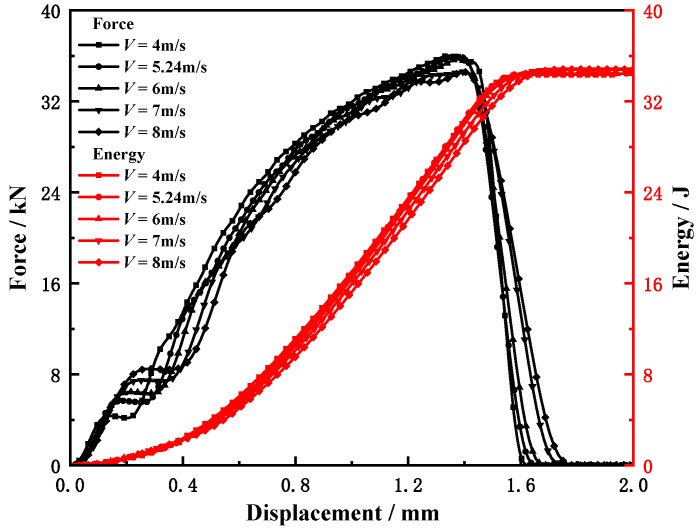
Influence of striker velocity on the load–displacement and energy–displacement curves.

The mechanical properties of the materials are strongly related to the deformation rate [33,47]. According to the test results in Section 2.2, it can be found that the mechanical properties of the material do not change much at low strain rates, while at high strain rates, the material properties change significantly. To study the influence of the striker velocity on the Charpy test of this material, based on the MJC model, JC failure model, and the determined parameters, the Charpy test was simulated with five striker velocities of 4, 5.24, 6, 7, and 8 m/s, and the effects of the different velocities on the energy and characteristic loads of each part were studied. The variations of the force and absorbed energy with displacement for different velocities are shown in Figure 15.

As shown in Figure 15, although the load–displacement curve is discrete in the ascending section, the trend is the same; there is also a certain discreteness in the descending section after reaching the maximum load. As the pendulum speed increases, the maximum load decreases. It can be seen from the energy displacement curve that in the ascending section before reaching the final platform, the slope decreases with the increase in the striking speed, but the final absorbed energy coincides; that is, the influence of the hammer speed on the impact result of the material can be ignored.

**Figure 16:**
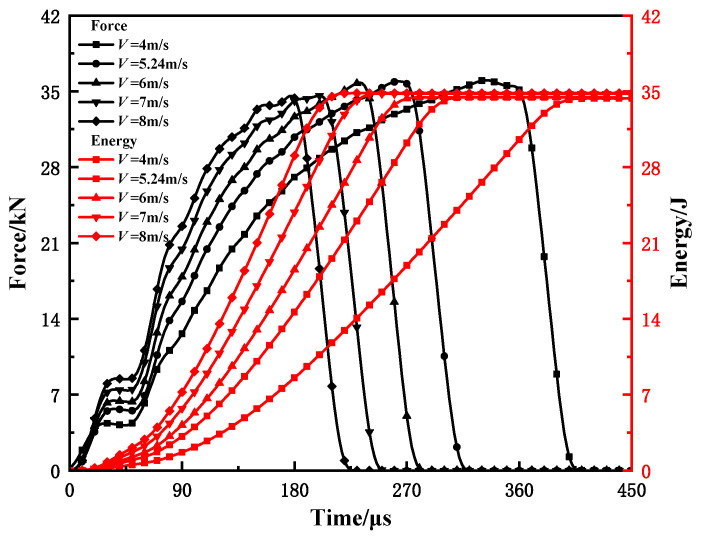
Influence of striker velocity on load–time and energy–time curves.

The variations of the force and absorbed energy with time at different velocities are shown in Figure 16. It can be seen that in the rising section, the load–time curve and the energy–time curve become steep with the increase in the impact speed. In addition, the absorbed energy is the same. This indicates that the hammer speed has a significant influence on the impact response process of the material.

**Figure 17:**
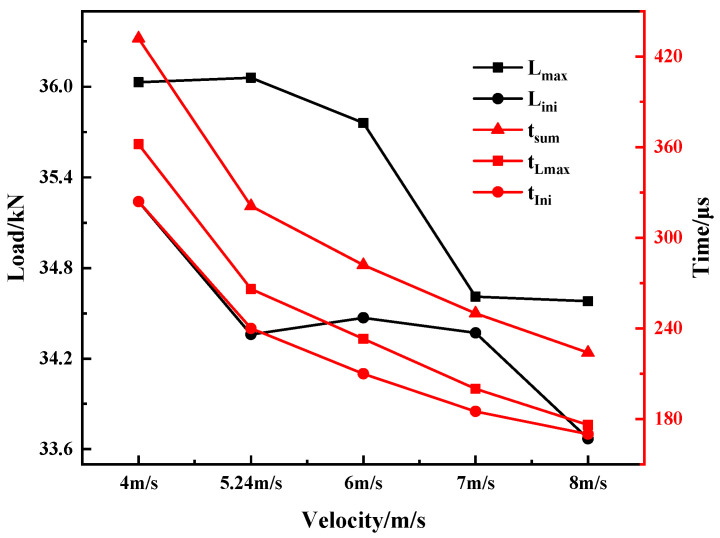
Characteristic time and load at different striker velocities.

According to the simulation results, the characteristic loads and the corresponding times of the samples under different strike speeds were obtained. The results are shown Table 5 and plotted in Figure 17. It can be seen that with the increase in the strike velocity, the maximum load time and crack initiation time all show a gradually decreasing and coinciding trend. This indicates that the hammer velocity has a significant influence on the response process of the sample.

**Figure 18:**
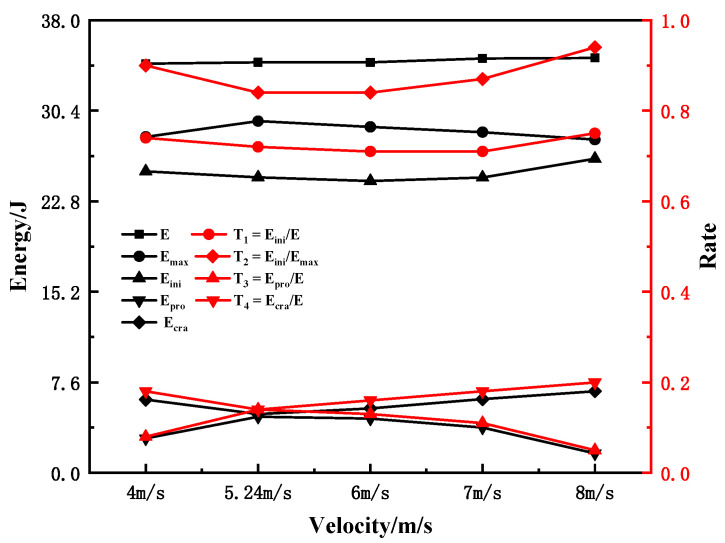
Characteristic energies and corresponding ratios at different striker velocities.

According to the simulation results, the energy of each part of the sample under different hammer velocities was obtained, and the ratio of each energy component to the total energy was calculated, as shown in Table 6 and plotted in Figure 18. It can be seen that *E*, *E_ini_*, *E_pro_*, *E_cra_*, and *E_max_* did not change significantly with the increase in strike speed. The rates of change of *E_max_* and *E_ini_* were within 6%. The change in *E* was within 1%. Under different hammer velocities, the changes of T_2_, and T_4_, were not significant.

The impact of hammer velocity on the total energy and the energy of each part absorbed by the 30CrMnSiNi2A high-strength steel Charpy impact test specimen was small, indicating that the impact of hammer velocity on the response results of the specimen can be ignored. However, with the increase in hammer speed, the maximum load, crack initiation load and its corresponding time were closer. This indicates that the structural integrity of the material used under dynamic conditions has an important relationship with the loading rate.

### 4.2. Effect of Specimen Width

For components with small section thicknesses or complex shapes, standard CVN cannot be processed. Sub-size Charpy specimens with reduced widths are usually selected for processing and testing [31,47]. Typical widths of SCVN are 7.5 mm (3/4 size), 6.66 mm (2/3 size), 5 mm (1/2 size), 3.33 mm (1/3 size), and 2.5 mm (1/4 size), respectively.

In this study, the CVN and SCVN samples were selected to study the effects of width on E_ini_, E_pro_, and E_cra_ and their corresponding characteristic values. According to the Charpy impact test conditions, the initial velocity of the hammer was 5.24 m/s, the mass was 21.9 kg, and the energy of the pendulum was 300 J.

**Figure 19:**
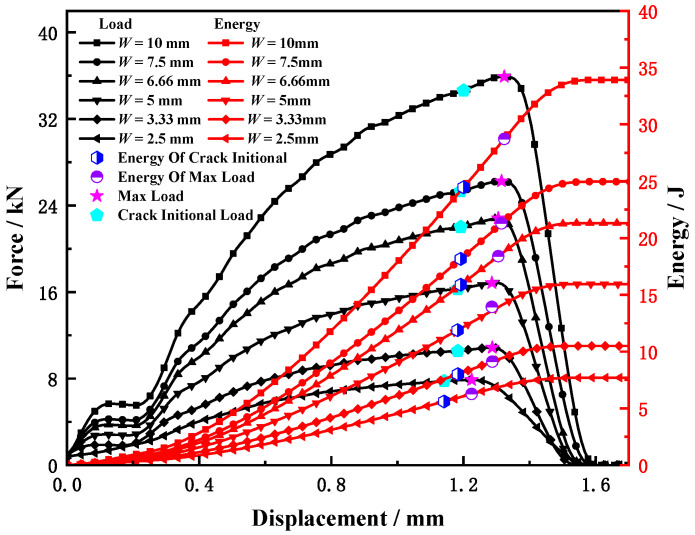
The load–displacement and energy–displacement curves for different widths.

The influence of sample width on the Charpy impact test results is shown in Figure 19. It can be seen that with the increase in specimen width, the displacement of the specimen hammered out of the anvil gradually increases, and the maximum load, crack initiation load and corresponding absorbed energy also gradually increase.

**Figure 20:**
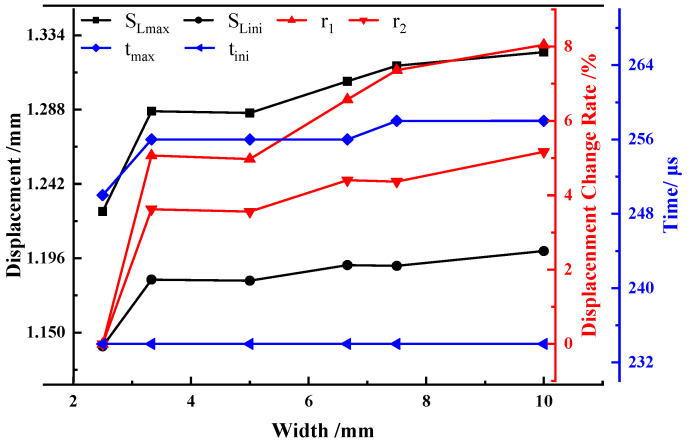
Characteristic time, load and displacement, corresponding ratio, and change rate.

**Figure 21:**
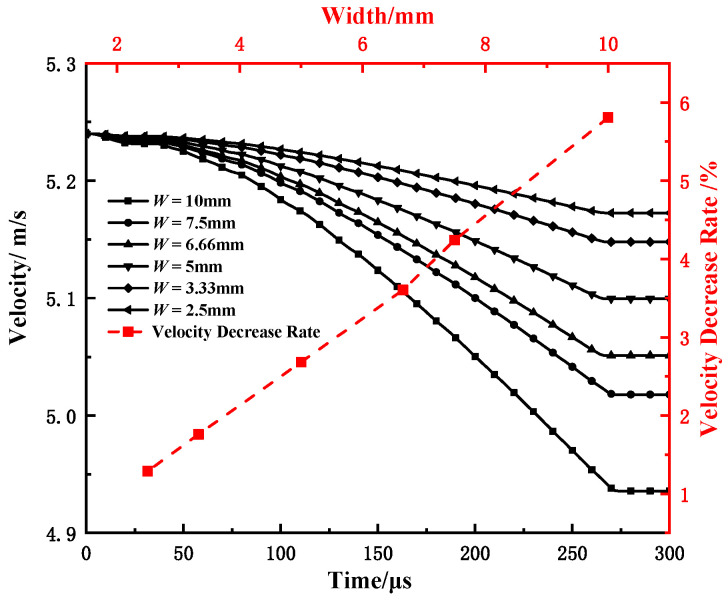
Effect of specimen width on velocity variations.

According to the simulation results, the time, load, and displacement corresponding to the characteristic points of the samples with different widths were extracted, and the corresponding change rates were calculated. The results are shown in Table 7 and plotted in Figure 20. It can be seen that with the increase in sample width, the crack initiation time does not change, and the maximum load time shows a trend of gradual increase. During the entire test process, the proportion of the absorbed energy by the sample to the pendulum energy was small, and the standard CVN sample was only 11.3%, and the hammer speed decrease was only 5.8%, indicating that the entire test process was carried out at a constant speed, as shown in Figure 21. This may be the main reason for the invariable crack initiation time and slight change in the maximum load point time.

With the increase in sample width, both the crack initiation displacement and maximum load displacement exhibited a trend of gradual increase. Compared with the 2.5 mm sample, the crack initiation displacement and maximum load displacement of the 10 mm sample increased by 8.05% and 5.17%, respectively. In other words, the sample width has little influence on the maximum load displacement, but it has a significant influence on the crack initiation displacement.

**Figure 22:**
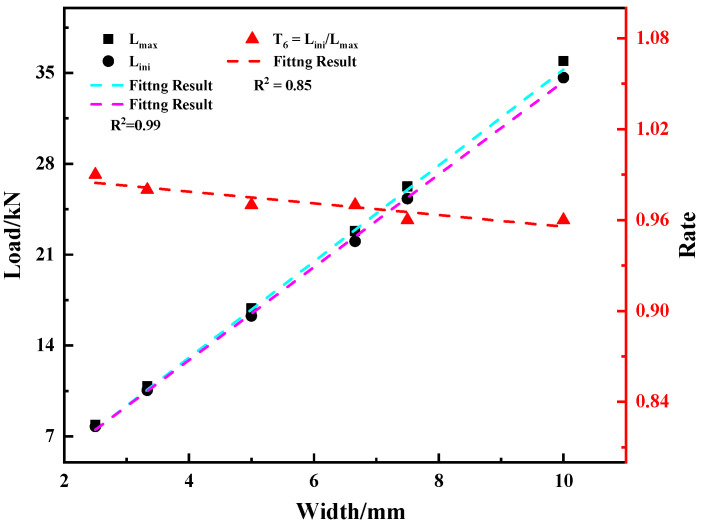
Variation of characteristic load and its ratio with sample width.

Figure 22 shows the variation trends of maximum load and crack initiation load with sample width. It can be seen that with the increase in sample width, the maximum load and crack initiation load show a linear increasing trend; and the difference between the initial crack load and the maximum load increases. The relationships between the maximum load, crack initiation load, and its ratio T_6_ with the sample width are shown in Equations (16)–(18).

Fmax = −1.735 + 3.7 *W* (16)

Fini = −1.419 + 3.575 *W* (17)

T6 = 0.9942 − 0.00386 *W* (18)

**Figure 23:**
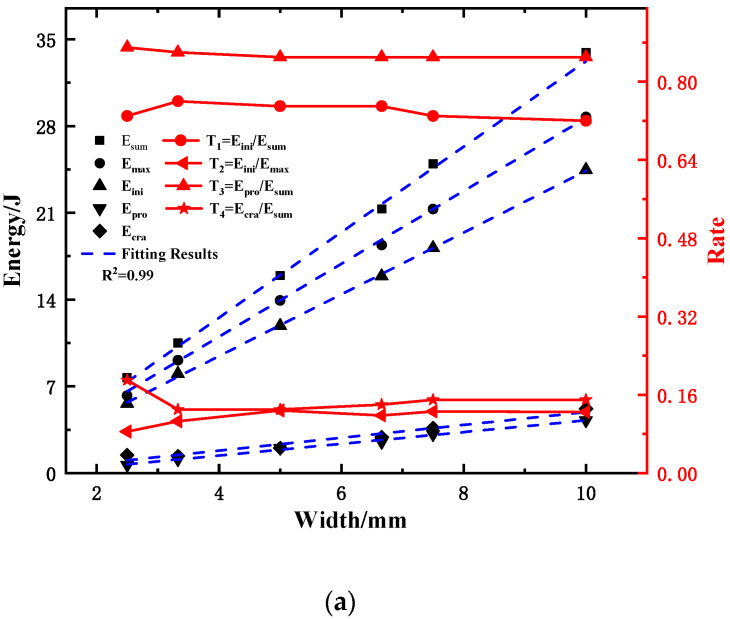

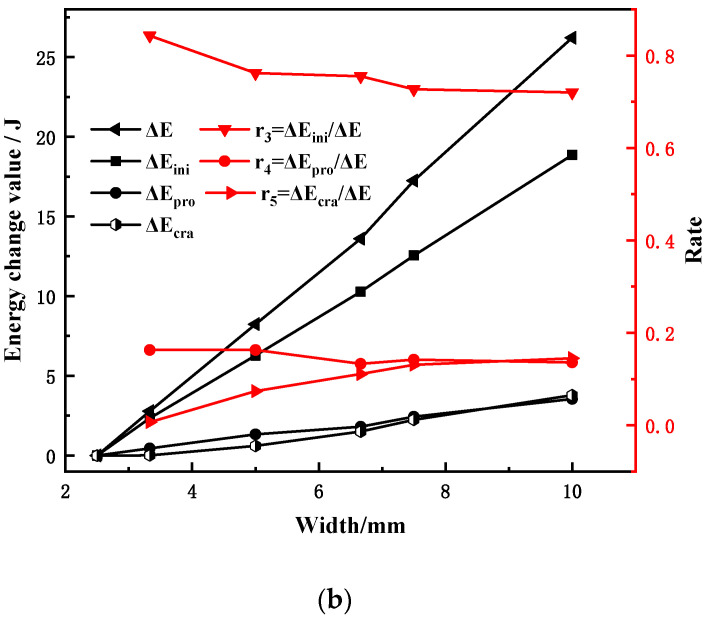
The variation of characteristic energy and corresponding ratio with sample width and fitting results. (**a**) the increment and proportion of the energy of each part; (**b**) the relative increment and proportion of the energy of each part.

The characteristic energy and corresponding ratio of the sample are shown in Table 8 and plotted in Figure 23a,b. From Figure 23a, it can be seen that E_ini_ accounts for more than 70% of the total energy, whereas E_pro_ and E_cra_ only account for approximately 10% each, indicating that the impact energy is mainly consumed at the crack initiation stage. This is well consistent with the results of studies [11,47]. The ability of materials to resist crack initiation and crack propagation is the key to ensuring structural integrity [11]. Therefore, in the material selection, comparing and analyzing the E_ini_ and E_pro_ of the material is the key to ensuring the accuracy of the material selection. Therefore, it is more reasonable to use E_ini_ and E_pro_ to evaluate the impact resistance of materials. With the increase in the sample width, E_ini_, E_pro_, and E_cra_ all show a linear increasing trend. However, the correlation between the energy of each part and the sample width is quite different; E_ini_ has a very strong correlation with the sample width, whereas those of E_pro_, and E_cra_ are weak. This shows that the increase in the sample width greatly improves the resistance of the material to crack initiation, while the improvement of the resistance to stable crack propagation is weak.

With the increase in the sample width, the T_1_ shows a decreasing trend, indicating that the increase in the crack initiation energy has an inverse correlation with the increase in the sample width. In addition, T_1_ is above 70%, indicating that the crack initiation energy dominates the increase in the total energy. T_2_ shows a decreasing trend, but it remains above 80%, indicating that it is unreasonable to set the maximum load position as the crack initiation point. This result is consistent with the research conclusions of Server [15], Toshiro [21], and Ray et al. [22]. The T_3_ shows a trend of gradually increasing and tending to be stable, indicating that the increase in the width of the sample improves the ability of the sample to resist stable crack propagation; however, when the width of the sample increased by four times, the T_3_ only increased by approximately 5%, with a small increase in capacity.

Figure 23b shows the increase in total energy and energy of each part with the sample thickness (relative to a 2.5 mm sample) and the proportion of the increase in each part energy in the increase in total energy. It can be seen that with the increase in sample thickness, ΔE_ini_ and ΔE show a strong correlation with the sample width, while the correlation between ΔE_pro_ and ΔE_cra_ and the sample width is weak. Meanwhile, r_3_ is over 70%, indicating that the crack initiation energy dominates in the increase in the total energy with the sample width. However, r_3_ and r_4_ show a gradually decreasing trend, and r_5_ shows a gradually increasing trend, indicating that the impact toughness of the Charpy sample gradually decreased with the increase in sample width. This is consistent with the conclusion of scholars [6,11,13] that the Charpy impact toughness decreases gradually with the increase in the sample size; however, previous studies did not analyze the internal reason that the impact toughness decreases with the sample size. Fortunately, the research results in this section reveal the inner mechanism of this result in depth.

The conversion relationship between the impact energy of SCVN and CVN specimens has been a major research subject [6,7,8,9,10,11,12]. Although the ASTM A370 [31] standard stipulates that the conversion relationship between them is proportional to the width, some studies have found that this law does not hold when the sample is small, and the proportion coefficient is different for different materials. According to the above research results, it can be found that the fundamental reason is that with the change of sample width, the energy variation law of each part is greatly different, and the energy increment of each part is not positively correlated with the sample width. Therefore, we believe that this is the fundamental reason for the failure of the linear relationship between samples of different sizes of absorbed energy. At the same time, it is very meaningful and necessary to divide Charpy impact energy and study the variation of energy of each part with sample width.

For the 30CrMnSiNi2A steel selected in this study, the linear relationship between the impact energy and the sample width was very good. However, to ensure the accuracy of the conversion and analysis between the impact energy of the SCVN and CVN samples, the relationship between the energy of each part and the sample was established, as shown in Equations (19)–(21). It can be observed that the linear relationship between the energy of each part and the width of the sample is completely different, and the difference is significant.

Eini = −1.2608 + 2.4898 *W* (19)

Epro = −0.2605 + 0.5188 *W* (20)

Ecra = −0.4746 + 0.4729 *W* (21)

## 5. Conclusions

In this study, the static and dynamic deformation behavior, the effect of the stress state on the fracture behavior, and Charpy impact properties of 30CrMnSiNi2A high-strength steel were studied through a large number of experiments. An MJC model was established to accurately describe the flow behavior and strain rate behavior of 30CrMnSiNi2A high-strength steel, and the MJC model and JC fracture model parameters were determined. The Charpy impact test was simulated, and the simulation results were in good agreement with the test results. The impact energy was decomposed based on the simulation results. On this basis, the influence of the striker speed and the sample width of the sample on the energy of each part (crack initiation energy, crack stable propagation energy, and crack unstable propagation energy) and characteristic loads (crack initiation load and maximum load) were studied. Finally, the influence of the sample width on the energy of each part was deeply analyzed. The influence mechanism by which Charpy impact toughness decreases with the increase in specimen size is discussed. The internal reason that the linear conversion relationship between the Charpy impact energy of the SCVN specimen and CVN specimen does not hold for small size specimens is analyzed and discussed. An impact test correlation model of SCVN specimen and CVN specimen was established. The following conclusions are obtained:

1. The MJC model was established, and the MJC model parameters (A = 1290 MPa, B = 595 MPa, w = 1.084, *n* = 0.01435, C_1_ = 0.0025, C_2_ = 0.029, C_3_ = 15.25) and JC fracture model parameters (D_1_ = −0.1663, D_2_ = 1.7969, D_3_ = −2.9078, D_4_ = 0.07) were determined. The instrumented Charpy test was simulated, and the simulation and test results were in good agreement. It is proven that the MJC model and JC fracture models can simulate the deformation behavior and failure characteristics of the material under dynamic conditions.
2. The influence of the pendulum speed on the energy and characteristic load of each part is small and can be ignored. However, it had a significant influence on the response of the Charpy specimen. The faster the pendulum speed, the shorter the time between the crack initiation load and the maximum load is, and the closer it is.
3. The width of the sample has a significant influence on the energy and characteristic load of each part, but the law of influence on the energy of each part is quite different; the linear correlation between the crack initiation energy and the sample width is very strong, whereas the linear correlations between the stable crack propagation energy and crack unstable propagation energy and the sample width are weak. With the increases in the width of the specimen, the difference between the stable crack propagation energy, the unstable crack propagation energy, and the crack initiation energy is larger.
4. Under the condition of different sample widths, more than 70% of the impact energy was consumed in the crack initiation stage. The ability of a material to resist crack initiation and resist stable crack propagation is the key to its resistance to fracture [11]. Therefore, in the selection of materials, comparison and analysis of the crack initiation energy and stable crack propagation energy can better ensure the accuracy of material selection.
5. With the increase in sample width, the increment of crack initiation energy and the increment of the crack stable growth energy decreased gradually. This discovery reveals that the internal mechanism of the Charpy impact toughness decreases with the increase in sample size. This finding also reveals the internal reason why the linear transformation relationship between the Charpy impact energy of SCVN specimens and CVN specimens is not tenable when the specimens are small. Because of the 30CrMnSiNi2A steel material selected in this study, the linear correlation between the SCVN specimens and CVN specimens was good. This finding needs to be confirmed through in-depth studies on various materials.
6. The energy of each part of the correlation model for the SCVN and CVN specimens in the impact test was established. Equations (19)–(21) can be used to convert the Charpy impact test results of any width to standard test results more accurately.

## Figures and Tables

**Table 1:** Chemical composition of 30CrMnSiNi2A steel.

C	Si	Mn	S	P	Ni	Cr	Mo	V	Cu	Ti	W
0.28	1.06	1.11	0.005	0.011	1.51	1.05	0.1	0.01	0.17	0.03	0.03

**Table 2:** Mechanical properties under different strain rates.

	Static State		Dynamic State			
strain rates	10^−3^ s^−1^	10^−1^ s^−1^	1300 s^−1^	1700 s^−1^	2000 s^−1^	2500 s^−1^
yield strength/MPa	1290	1300	1790	1850	1900	2000
ultimate strength/MPa	1916	1905				

**Table 3:** Facture strain and stress triaxiality of specimens.

R/mm	*r*/mm	$r_{f}/\mathrm{mm}$	$\varepsilon_{f}\;$	$\sigma^{*}$	
				Model	Simulation
3	2	1.875	0.129	0.62	0.62
4	2	1.815	0.194	0.56	0.56
9	2	1.700	0.325	0.44	0.42

**Table 4:** Comparison of Charpy test and simulation results.

	Experiment Data	Simulation Data	Error
Maximum load/kN	37.39	38.13	1.98%
Impact energy/J	36.33	35.68	−1.79%

**Table 5:** Characteristic time and load at different hammer velocities.

V/m/s	t_sum_/μs	t_Lsmax_/μs	t_Ini_/μs	L_max_/kN	L_ini_/kN
4	432	362	324	36.03	35.24
5.24	321	266	240	36.06	34.36
6	282	233	210	35.76	34.47
7	250	200	185	34.61	34.37
8	224	176	170	34.58	33.67

**Table 6:** Characteristic energies and corresponding ratios at different striker velocities.

*V*/m/s	*E*/J	*E_max_*/J	*E_ini_*/J	*E_pro_*/J	*E_cra_*/J	T_1_	T_2_	T_3_	T_4_
4	34.35	28.20	25.31	9.04	6.15	0.74	0.90	0.08	0.18
5.24	34.46	29.52	24.81	9.65	4.94	0.72	0.84	0.14	0.14
6	34.45	29.04	24.49	9.96	5.41	0.71	0.84	0.13	0.16
7	34.79	28.60	24.80	9.99	6.19	0.71	0.87	0.11	0.18
8	34.83	27.98	26.36	8.48	6.85	0.75	0.94	0.05	0.20

**Table 7:** Characteristic time, load and displacement, corresponding ratio and change rate.

W /mm	t_max_ /μs	t_ini_ /μs	L_max_ /kN	L_ini_ /kN	S_Lmax_ /mm	S_Lini_ /mm	t_1_	r_1_ /%	r_2_ /%
10	258	234	35.90	34.53	1.32	1.20	0.96	5.17	8.05
7.5	258	234	26.26	25.32	1.32	1.19	0.96	4.37	7.36
6.66	256	234	22.78	22.02	1.31	1.19	0.97	4.47	6.58
5	256	234	16.85	16.28	1.29	1.18	0.97	3.56	4.97
3.33	256	234	10.73	10.55	1.29	1.18	0.97	3.62	5.07
2.5	250	234	7.85	7.77	1.23	1.14	0.98	0.00	0.00

**Table 8:** Characteristic energies and corresponding ratios of samples with different widths.

W/mm	E/J	E_max_/J	E_ini_/J	E_pro_/J	E_cra_/J	ΔE/J	ΔE_ini_/J	ΔE_pro_/J	ΔE_cra_/J
10	33.92	29.08	24.13	4.25	5.19	26.21	18.86	3.55	3.79
7.5	24.96	21.30	18.16	3.14	3.65	17.25	12.55	2.44	2.25
6.66	21.31	18.63	15.89	2.51	2.91	13.60	10.28	1.81	1.51
5	15.94	13.93	11.89	2.04	2.01	8.24	6.28	1.34	0.61
3.33	10.49	9.34	7.96	1.11	1.42	2.79	2.35	0.45	0.02
2.5	7.71	6.42	5.66	0.70	1.40	0.00	0.00	0.00	0.00
**W/mm**	**T_1_**	**T_2_**	**T_3_**	**T_4_**	**r_3_**	**r_4_**	**r_5_**		
10	0.72	0.83	0.13	0.15	0.720	0.136	0.145		
7.5	0.73	0.85	0.12	0.15	0.727	0.142	0.131		
6.66	0.75	0.85	0.11	0.14	0.755	0.133	0.111		
5	0.75	0.85	0.12	0.13	0.762	0.163	0.074		
3.33	0.76	0.86	0.11	0.13	0.843	0.163	0.007		
2.5	0.74	0.87	0.08	0.18	0.00	0.00	0.00		

## Data Availability

The data presented in this study are available on request from the corresponding author.

## Nomenclature

A	initial yield stress of materials at reference strain rate and reference temperature
B	strain hardening modulus
C_1_, C_2_	the strain rate-hardening parameter
C_3_	the natural logarithm of the critical strain rate level
CVN	full-size Charpy V-notch specimens
D_1_, D_2_, D_3_, D_4_,	the material damage parameters
E	impact energy
E_max_	maximum load energy
E_ini_	crack initiation energy
E_pro_	crack stable propagation energy
E_cra_	crack instability propagation energy
ΔE	increase in impact energy
ΔE_ini_	increase in crack initiation energy
ΔE_pro_	increase in crack stable propagation energy
ΔE_cra_	increase in crack instability propagation energy
L_max_	maximum load
L_ini_	crack initiation load
r_1_	the change rate of maximum load displacement
r_2_	the change rate of crack initiation displacement
r_3_	the rate of ΔE_ini_/ΔE
r_4_	the rate of ΔE_pro_/ΔE
r_5_	the rate of ΔE_cra_/ΔE
*ε*	engineering strain
*ε*_f_	fracture strain
*ε*_p_	plastic strain
*ε*_t_	true stain
${\dot{\varepsilon }}^{*}$	dimensionless strain rate
${\dot{\varepsilon }}_{0}$	reference strain rate
*σ*	engineering stress
*σ*_eq_	equivalent flow stress
*σ*_m_	mean stress or hydrostatic stress
*σ*_t_	true stress
*σ* ^∗^	stress triaxiality
SCVN	sub-size Charpy V-notch specimens
S_Lini_	crack initiation displacement
S_Lmax_	maximum load displacement
T	working temperature
T_0_	room temperature
T_r_	melting temperature
t_Ini_	crack initiation time
t_sum_	total time
t_Lmax_	maximum load time
T_1_	the ratio of crack initiation energy to total energy
T_2_	the ratio of crack initiation energy to maximum load energy
T_3_	the ratio of the crack stable propagation energy to the total energy
T_4_	the ratio of crack instability propagation energy to total energy
t_1_	the ratio of crack initiation load to max load
w, n	strain hardening index parameters
W	width
